# Crystal structure of [butane-2,3-dione bis­(4-methyl­thio­semicarbazonato)-κ^4^ *S*,*N* ^1^,*N* ^1′^,*S*′](pyridine-κ*N*)zinc(II)

**DOI:** 10.1107/S2056989015019234

**Published:** 2015-10-17

**Authors:** Oliver C. Brown, Derek A. Tocher, Philip J. Blower, Michael J. Went

**Affiliations:** a University of Kent, School of Physical Sciences, Canterbury CT2 7NH, England; b Department of Chemistry, University College London, 20 Gordon Street, London WC1H 0AJ, England; c King’s College London, Division of Imaging Sciences and Biomedical Engineering, 4th Floor Lambeth Wing, St Thomas’ Hospital, London SE1 7EH, England

**Keywords:** crystal structure, bis­(thio­semicarbazone), copper, zinc, hypoxia, PET

## Abstract

In the structure of the title complex, [Zn(C_8_H_14_N_6_S_2_)(C_5_H_5_N)], the Zn^II^ ion has a pseudo-square-pyramidal coordination environment and is displaced by 0.490 Å from the plane of best fit defined by the bis­(thio­semicarbazonate) N_2_S_2_ donor atoms. Chains sustained by intermolecular N—H⋯N and N—H⋯S hydrogen-bonding interactions extend parallel to [10-1].

## Abstract 2

The title compound was prepared by the reaction of [butane-2,3-dione bis­(4-methyl­thio­semicarbazonato)]zinc(II) with pyridine. The Zn^II^ atom is five-coordinate in a pseudo-square-pyramidal geometry.

## Chemical context   

Bis(thio­semicarbazonato)copper complexes labelled with ^60/62/64^Cu isotopes are useful radiopharmaceuticals for imaging blood flow and hypoxic tissues *in vivo* (Dearling *et al.*, 2002 ▸). Bis(thio­semicarbazonato)zinc complexes can act as precursors for bis­(thio­semicarbazonato)copper complexes by reaction with copper acetate in water (Holland *et al.*, 2007 ▸). This synthetic approach can be very useful in the quick, clean synthesis of radio-copper complexes, particularly if the copper isotope has a short half live. A solid-phase synthesis has been developed based on the attachment of a bis­(thio­semi­carba­zonato)zinc complex to 4-(di­methyl­amino)­pyridine function­al­ized polystyrene resin and elution of the desired radio-copper complex by the addition of a [^64^Cu]copper acetate solution (Betts *et al.*, 2008 ▸). A number of different polymers for zinc–copper bis­(thio­semicarbazonato) transmetalation reactions have been tested and a pyridyl system was found to be optimal (Aphaiwong *et al.*, 2012 ▸). This communication reports the crystal structure of a zinc bis­(thio­semi­carbazonato) pyridine complex, [Zn(C_8_H_14_N_6_S_2_)(C_5_H_5_N)]. Comparison of the infra-red and Raman spectra indicates that [butane-2,3-dione bis­(4-methyl­thio­semi­carbazonato)]zinc(II) coordinates to poly(4-vinyl­pyri­dine) (Brown 2015 ▸). 
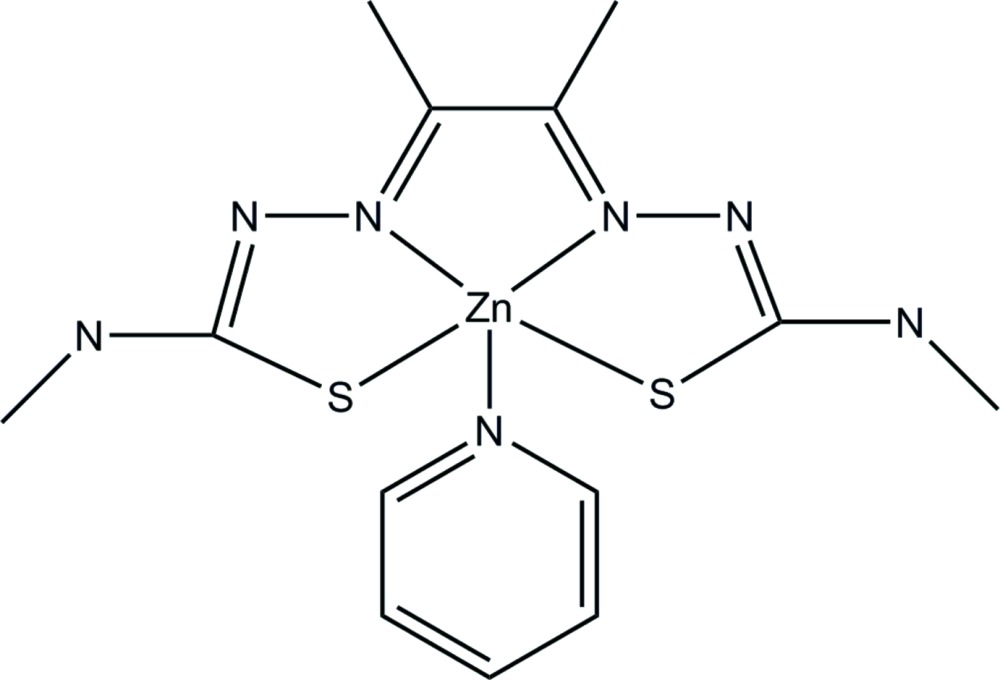


## Structural commentary   

**Figure 1:**
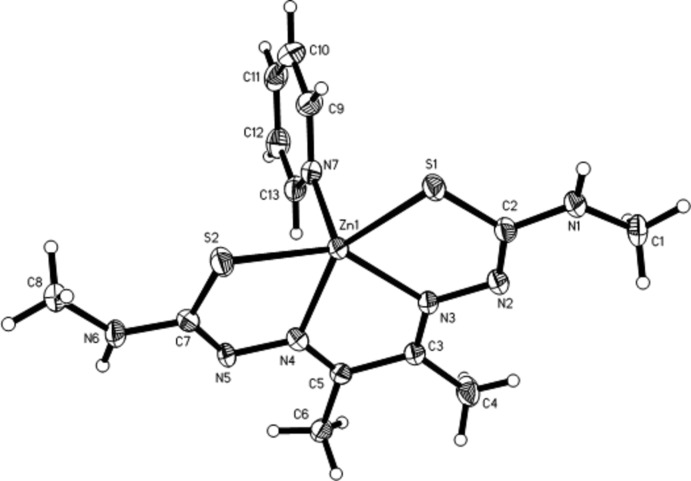
The mol­ecular structure of the title complex.

The molecular structure of [butane-2,3-dione bis­(4-methyl­thio­semicarbazonato)]pyridine­zinc is shown in Fig. 1 ▸. The Zn^II^ ion lies in a pseudo-square-pyramidal coord­ination and is displaced by 0.490 Å from the plane of best fit defined by the bis­(thio­semicarbazonate) N_2_S_2_ donor atoms. In the related 4-(di­methyl­amino)­pyridine complex, the displace­ment is 0.517 Å (Betts *et al.*, 2008 ▸). The Zn–pyridine bond is shorter [2.0900 (18) Å] than the other two bonds to atoms N3 and N4. It is apparent that the ligand cavity is too small to fit the Zn^II^ ideally, resulting in an N—Zn—N angle of only 74.45 (7)° which may contribute to the ready transmetalations that result in Cu^II^ complexes with angles of approximately 80° (Blower *et al.*, 2003 ▸). A comparison of the vibrational spectroscopy of poly(4-vinylpyridine), [butane-2,3-dione bis(4-methylthiosemicarbazonato)]zinc(II) and [butane-2,3-dione bis(4-methylthiosemicarbazonato)]zinc(II) on poly(4-vinylpyridine) can be found in the supporting information.

## Supramolecular features   

**Figure 2:**
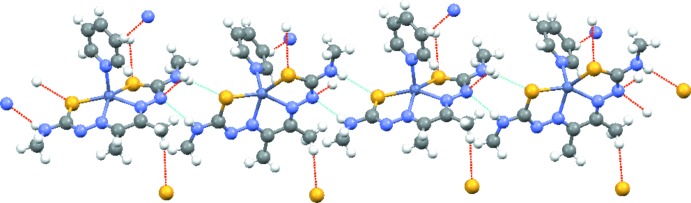
The chain structure of the title complex formed by N—H⋯N and N—H⋯S hydrogen bonds. The chain direction is parallel to [10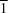].

The mol­ecules form a chain *via* N6—H6⋯S1 (2.65 Å) and N1—H1⋯N5 (2.21 Å) hydrogen bonds (Table 1 ▸), as has been seen previously in related Cu^II^ bis­(thio­semicarbazonate) complexes (Blower *et al.*, 2003 ▸), with weaker inter­actions between the chains [H6*A*⋯S2(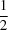 + *x*, 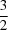 − *y*, 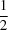 + *z*) = 2.88 Å and H12⋯N5(−*x*, 1 − *y*, 1 − *z*) = 2.67 Å] (see Fig. 2 ▸).

## Synthesis and crystallization   

[Butane-2,3-dione bis­(4-methyl­thio­semicarbazonato)]zinc (0.194 g, 0.60 mmol) was dissolved in DMSO (2 ml). Pyridine (0.06 ml, 0.059 g, 0.70 mol) was added to the solution and left to stir overnight. Water (5 ml) was added to solution. The crystalline precipitate was recovered *via* filtration, washed with ethanol (1 × 10 ml) and diethyl ether (5 × 10 ml). The solid was dried in air. A yellow solid (0.125 g) was recovered (52% yield). 

## Spectroscopic data   

^1^H NMR (DMSO-*d*_6_, 400 MHz): δ 8.49 (2H, *m*, H_(2,6)_ pyrid­yl), 7.79 (2H, *m*, H_(4)_ pyrid­yl), 7.39 (2H, *m*, H_(3,5)_ pyrid­yl), 7.18 (2H, *s*, H_3_C-N*H*), 2.79 (6H, *m*, HN-C*H*_3_), 2.26 (6H, *s*, N=C—C*H*_3_). ^13^C {^1^H} NMR (DMSO-*d*_6_, 100 MHz): δ 149.72 (C_(2,6)_ pyrid­yl), 137.57 (C_(4)_ pyrid­yl), 124.90 (C_(3,5)_ pyrid­yl), 29.81 (HN—CH_3_), 14.47 (N=C—*C*H_3_). IR (cm^−1^) 3273 (*w*), 3217 (*w*), 3001 (*w*), 2938 (*w*), 1603 (*w*), 1530 (*m*), 1510 (*m*), 1476 (*m*), 1447 (*m*), 1396 (*m*), 1337 (*m*), 1250 (*s*), 1213 (*s*), 1157 (*m*), 1072 (*s*), 1040 (*s*), 1013 (*m*), 974 (*m*), 839 (*m*), 760 (*m*), 694 (*s*), 648 (*m*), 635 (m), 590 (*m*), 446 (*s*). Raman (632.81 nm): cm^−1^ = 3285 (*w*), 1613 (*w*), 1544 (*s*), 1513 (*s*), 1478 (*m*), 1377 (*w*), 1337 (*w*), 1285 (*m*), 1254 (*m*), 1217 (*w*), 1190 (*w*), 1037 (*w*), 1013 (*w*), 989 (*w*),841 (*w*), 795 (*w*), 726 (*w*), 592 (*w*), 538 (*w*), 448 (*w*), 375 (*w*), 334 (*w*), 289 (*w*). Found for Zn_1_C_13_H_19_N_7_S_2_: C, 38.8; H, 4.6; N, 24.3. Calculated for Zn_1_C_13_H_19_N_7_S_2_: C, 38.8; H, 4.75; N, 24.3%. UV–Vis: λ_max_/nm (DMSO) 314 (∊/dm^3^ mol^−1^ cm^−1^ 12 600) and 434 (12 800).

## Refinement   

Crystal data, data collection and structure refinement details are summarized in Table 2 ▸. Hydrogen atoms were included in idealized positions and refined as riding: N—H = 0.86 Å, C—H = 0.93 (aromatic) or 0.96 (meth­yl) Å; *U*_iso_(H) = 1.2*U*_eq_(C,N) or 1.5*U*_eq_(C_meth­yl_). Methyl H atoms were generated in idealized positions and refined as rotating groups. [please check added text]

## Supplementary Material

Crystal structure: contains datablock(s) I. DOI: 10.1107/S2056989015019234/pj2023sup1.cif

Structure factors: contains datablock(s) I. DOI: 10.1107/S2056989015019234/pj2023Isup2.hkl

Supporting information file. DOI: 10.1107/S2056989015019234/pj2023sup3.pdf

CCDC reference: 1430734

Additional supporting information:  crystallographic information; 3D view; checkCIF report

## Figures and Tables

**Table 1:** Hydrogen-bond geometry (, )

*D*H*A*	*D*H	H*A*	*D* *A*	*D*H*A*
N1H1N5^i^	0.86	2.21	2.988(3)	150
N6H6S1^ii^	0.86	2.65	3.500(2)	167

Symmetry codes: (i) 
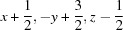
; (ii) 
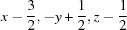
.

**Table 2:** Experimental details

Crystal data	
Chemical formula	[Zn(C_8_H_14_N_6_S_2_)(C_5_H_5_N)]
*M*_r_	402.84
Crystal system, space group	Monoclinic, *P*2_1_/*n*
Temperature (K)	150
*a*, *b*, *c* ()	10.1466(2), 13.9076(3), 12.7775(3)
()	104.756(2)
*V* (^3^)	1743.64(7)
*Z*	4
Radiation type	Cu *K*
(mm^1^)	4.27
Crystal size (mm)	0.26 0.04 0.02

Data collection	
Diffractometer	Agilent SuperNova Dual Source diffractometer with an Atlas detector
Absorption correction	Multi-scan (*CrysAlis PRO*; Agilent, 2014 ▸)
*T*_min_, *T*_max_	0.775, 1.000
No. of measured, independent and observed [*I* > 2(*I*)] reflections	12050, 3445, 3020
*R*_int_	0.041
(sin /)_max_ (^1^)	0.622

Refinement	
*R*[*F* ^2^ > 2(*F* ^2^)], *wR*(*F* ^2^), *S*	0.031, 0.074, 1.04
No. of reflections	3445
No. of parameters	212
H-atom treatment	H-atom parameters constrained
_max_, _min_ (e ^3^)	0.36, 0.42

Computer programs: (*CrysAlis PRO*; Agilent, 2014 ▸), *SHELXS97* (Sheldrick, 2008 ▸), *SHELXL2014* (Sheldrick, 2015 ▸) and *OLEX2* (Dolomanov *et al.*, 2009 ▸).

## supplementary crystallographic information

### Crystal data

**Table:**

[Zn(C_8_H_14_N_6_S_2_)(C_5_H_5_N)]	*F*(000) = 832
*M**_r_* = 402.84	*D*_x_ = 1.535 Mg m^−^^3^
Monoclinic, *P*2_1_/*n*	Cu *K*α radiation, λ = 1.54184 Å
*a* = 10.1466 (2) Å	Cell parameters from 6492 reflections
*b* = 13.9076 (3) Å	θ = 4.8–73.0°
*c* = 12.7775 (3) Å	µ = 4.27 mm^−^^1^
β = 104.756 (2)°	*T* = 150 K
*V* = 1743.64 (7) Å^3^	Needle, clear yellow
*Z* = 4	0.26 × 0.04 × 0.02 mm

### Data collection

**Table:**

Agilent SuperNova Dual Source diffractometer with an Atlas detector	3445 independent reflections
Radiation source: sealed X-ray tube, SuperNova (Cu) X-ray Source	3020 reflections with *I* > 2σ(*I*)
Mirror monochromator	*R*_int_ = 0.041
Detector resolution: 5.2031 pixels mm^-1^	θ_max_ = 73.6°, θ_min_ = 4.8°
ω scans	*h* = −7→12
Absorption correction: multi-scan (*CrysAlis PRO*; Agilent, 2014)	*k* = −16→17
*T*_min_ = 0.775, *T*_max_ = 1.000	*l* = −15→15
12050 measured reflections	

### Refinement

**Table:**

Refinement on *F*^2^	Primary atom site location: structure-invariant direct methods
Least-squares matrix: full	Hydrogen site location: inferred from neighbouring sites
*R*[*F*^2^ > 2σ(*F*^2^)] = 0.031	H-atom parameters constrained
*wR*(*F*^2^) = 0.074	*w* = 1/[σ^2^(*F*_o_^2^) + (0.032*P*)^2^ + 1.1359*P*] where *P* = (*F*_o_^2^ + 2*F*_c_^2^)/3
*S* = 1.04	(Δ/σ)_max_ = 0.001
3445 reflections	Δρ_max_ = 0.36 e Å^−^^3^
212 parameters	Δρ_min_ = −0.42 e Å^−^^3^
0 restraints	

### Special details

**Table:**

Experimental. Absorption correction: CrysAlisPro, Agilent Technologies, Version 1.171.37.34 (release 22-05-2014 CrysAlis171 .NET) (compiled May 22 2014,16:03:01) Empirical absorption correction using spherical harmonics, implemented in SCALE3 ABSPACK scaling algorithm.
Geometry. All e.s.d.'s (except the e.s.d. in the dihedral angle between two l.s. planes) are estimated using the full covariance matrix. The cell e.s.d.'s are taken into account individually in the estimation of e.s.d.'s in distances, angles and torsion angles; correlations between e.s.d.'s in cell parameters are only used when they are defined by crystal symmetry. An approximate (isotropic) treatment of cell e.s.d.'s is used for estimating e.s.d.'s involving l.s. planes.

### Fractional atomic coordinates and isotropic or equivalent isotropic displacement parameters (Å^2^)

**Table:**

	*x*	*y*	*z*	*U*_iso_*/*U*_eq_	
Zn1	0.30020 (3)	0.73515 (2)	0.47000 (2)	0.01926 (9)	
S1	0.44685 (5)	0.79566 (4)	0.36852 (4)	0.02284 (12)	
S2	0.14259 (6)	0.85069 (4)	0.50133 (4)	0.02449 (13)	
N1	0.68465 (19)	0.71828 (14)	0.37359 (15)	0.0248 (4)	
H1	0.6813	0.7600	0.3233	0.030*	
N2	0.59011 (19)	0.65189 (13)	0.50031 (15)	0.0235 (4)	
N3	0.47831 (18)	0.65526 (13)	0.54215 (14)	0.0203 (4)	
N4	0.26884 (18)	0.68075 (13)	0.61711 (14)	0.0213 (4)	
N5	0.15519 (18)	0.70523 (13)	0.65027 (14)	0.0215 (4)	
N6	−0.02309 (19)	0.80647 (14)	0.62535 (15)	0.0257 (4)	
H6	−0.0411	0.7758	0.6785	0.031*	
N7	0.17142 (18)	0.64416 (13)	0.35929 (14)	0.0213 (4)	
C1	0.8028 (2)	0.65595 (18)	0.4039 (2)	0.0301 (5)	
H1A	0.8662	0.6725	0.3624	0.045*	
H1B	0.7748	0.5903	0.3898	0.045*	
H1C	0.8457	0.6638	0.4796	0.045*	
C2	0.5800 (2)	0.71402 (15)	0.42040 (17)	0.0214 (4)	
C3	0.4771 (2)	0.60479 (15)	0.62697 (16)	0.0211 (4)	
C4	0.5913 (3)	0.54130 (19)	0.6841 (2)	0.0344 (6)	
H4A	0.5647	0.4752	0.6712	0.052*	
H4B	0.6127	0.5542	0.7604	0.052*	
H4C	0.6700	0.5537	0.6575	0.052*	
C5	0.3557 (2)	0.61910 (15)	0.66955 (16)	0.0200 (4)	
C6	0.3438 (2)	0.56830 (17)	0.76987 (17)	0.0251 (4)	
H6A	0.4086	0.5947	0.8314	0.038*	
H6B	0.3618	0.5010	0.7639	0.038*	
H6C	0.2533	0.5766	0.7788	0.038*	
C7	0.0907 (2)	0.78126 (15)	0.59668 (17)	0.0215 (4)	
C8	−0.1183 (2)	0.88087 (17)	0.57441 (18)	0.0269 (5)	
H8A	−0.1695	0.8591	0.5046	0.040*	
H8B	−0.0689	0.9381	0.5662	0.040*	
H8C	−0.1794	0.8946	0.6188	0.040*	
C9	0.1317 (2)	0.67116 (17)	0.25522 (18)	0.0264 (5)	
H9	0.1650	0.7287	0.2350	0.032*	
C10	0.0434 (2)	0.61722 (18)	0.17651 (19)	0.0313 (5)	
H10	0.0177	0.6381	0.1050	0.038*	
C11	−0.0057 (2)	0.53150 (17)	0.2068 (2)	0.0301 (5)	
H11	−0.0652	0.4937	0.1558	0.036*	
C12	0.0346 (2)	0.50294 (17)	0.3136 (2)	0.0301 (5)	
H12	0.0030	0.4455	0.3356	0.036*	
C13	0.1226 (2)	0.56104 (16)	0.38725 (18)	0.0247 (4)	
H13	0.1492	0.5417	0.4592	0.030*	

### Atomic displacement parameters (Å^2^)

**Table:**

	*U*^11^	*U*^22^	*U*^33^	*U*^12^	*U*^13^	*U*^23^
Zn1	0.01595 (15)	0.02595 (15)	0.01686 (14)	0.00116 (10)	0.00599 (10)	0.00201 (10)
S1	0.0201 (3)	0.0283 (3)	0.0226 (2)	0.00170 (19)	0.0101 (2)	0.00528 (19)
S2	0.0234 (3)	0.0268 (3)	0.0264 (3)	0.0057 (2)	0.0122 (2)	0.0057 (2)
N1	0.0194 (9)	0.0345 (10)	0.0227 (9)	0.0000 (7)	0.0097 (7)	0.0049 (7)
N2	0.0193 (9)	0.0316 (10)	0.0219 (8)	0.0020 (7)	0.0095 (7)	0.0024 (7)
N3	0.0158 (9)	0.0268 (9)	0.0191 (8)	0.0023 (7)	0.0060 (7)	0.0020 (7)
N4	0.0202 (9)	0.0270 (9)	0.0186 (8)	0.0011 (7)	0.0081 (7)	0.0002 (7)
N5	0.0149 (9)	0.0318 (9)	0.0201 (8)	0.0022 (7)	0.0088 (7)	0.0010 (7)
N6	0.0182 (9)	0.0365 (10)	0.0246 (9)	0.0064 (8)	0.0098 (7)	0.0051 (8)
N7	0.0159 (9)	0.0259 (9)	0.0221 (8)	0.0001 (7)	0.0050 (7)	−0.0005 (7)
C1	0.0169 (11)	0.0405 (13)	0.0358 (12)	0.0037 (9)	0.0118 (9)	0.0010 (10)
C2	0.0176 (10)	0.0268 (10)	0.0209 (10)	−0.0029 (8)	0.0069 (8)	−0.0028 (8)
C3	0.0178 (10)	0.0270 (10)	0.0192 (9)	0.0017 (8)	0.0060 (8)	0.0019 (8)
C4	0.0292 (13)	0.0448 (14)	0.0331 (12)	0.0165 (11)	0.0149 (10)	0.0157 (11)
C5	0.0179 (10)	0.0257 (10)	0.0165 (9)	−0.0013 (8)	0.0047 (8)	−0.0002 (8)
C6	0.0185 (11)	0.0355 (12)	0.0210 (10)	0.0003 (9)	0.0047 (8)	0.0051 (9)
C7	0.0183 (11)	0.0295 (11)	0.0171 (9)	−0.0006 (8)	0.0055 (8)	−0.0036 (8)
C8	0.0209 (11)	0.0346 (12)	0.0263 (11)	0.0062 (9)	0.0077 (9)	−0.0014 (9)
C9	0.0230 (12)	0.0300 (11)	0.0247 (11)	−0.0010 (9)	0.0034 (9)	0.0025 (9)
C10	0.0255 (12)	0.0408 (13)	0.0242 (11)	0.0011 (10)	0.0004 (9)	0.0004 (9)
C11	0.0203 (11)	0.0334 (12)	0.0346 (12)	−0.0013 (9)	0.0032 (9)	−0.0096 (10)
C12	0.0240 (12)	0.0258 (11)	0.0410 (13)	−0.0018 (9)	0.0094 (10)	−0.0021 (10)
C13	0.0197 (11)	0.0274 (11)	0.0280 (11)	0.0017 (9)	0.0080 (8)	0.0037 (9)

### Geometric parameters (Å, º)

**Table:**

Zn1—S1	2.3635 (6)	C1—H1C	0.9600
Zn1—S2	2.3718 (6)	C3—C4	1.491 (3)
Zn1—N3	2.1218 (18)	C3—C5	1.482 (3)
Zn1—N4	2.1241 (17)	C4—H4A	0.9600
Zn1—N7	2.0900 (18)	C4—H4B	0.9600
S1—C2	1.760 (2)	C4—H4C	0.9600
S2—C7	1.738 (2)	C5—C6	1.495 (3)
N1—H1	0.8600	C6—H6A	0.9600
N1—C1	1.450 (3)	C6—H6B	0.9600
N1—C2	1.347 (3)	C6—H6C	0.9600
N2—N3	1.372 (3)	C8—H8A	0.9600
N2—C2	1.321 (3)	C8—H8B	0.9600
N3—C3	1.294 (3)	C8—H8C	0.9600
N4—N5	1.369 (2)	C9—H9	0.9300
N4—C5	1.288 (3)	C9—C10	1.385 (3)
N5—C7	1.337 (3)	C10—H10	0.9300
N6—H6	0.8600	C10—C11	1.385 (4)
N6—C7	1.344 (3)	C11—H11	0.9300
N6—C8	1.452 (3)	C11—C12	1.379 (4)
N7—C9	1.341 (3)	C12—H12	0.9300
N7—C13	1.341 (3)	C12—C13	1.382 (3)
C1—H1A	0.9600	C13—H13	0.9300
C1—H1B	0.9600		
			
S1—Zn1—S2	113.40 (2)	C5—C3—C4	120.98 (18)
N3—Zn1—S1	80.75 (5)	C3—C4—H4A	109.5
N3—Zn1—S2	144.85 (5)	C3—C4—H4B	109.5
N3—Zn1—N4	74.45 (7)	C3—C4—H4C	109.5
N4—Zn1—S1	150.39 (5)	H4A—C4—H4B	109.5
N4—Zn1—S2	80.36 (5)	H4A—C4—H4C	109.5
N7—Zn1—S1	102.52 (5)	H4B—C4—H4C	109.5
N7—Zn1—S2	101.09 (5)	N4—C5—C3	114.89 (18)
N7—Zn1—N3	107.07 (7)	N4—C5—C6	124.51 (19)
N7—Zn1—N4	100.05 (7)	C3—C5—C6	120.51 (18)
C2—S1—Zn1	95.38 (7)	C5—C6—H6A	109.5
C7—S2—Zn1	94.57 (7)	C5—C6—H6B	109.5
C1—N1—H1	118.4	C5—C6—H6C	109.5
C2—N1—H1	118.4	H6A—C6—H6B	109.5
C2—N1—C1	123.16 (19)	H6A—C6—H6C	109.5
C2—N2—N3	111.67 (18)	H6B—C6—H6C	109.5
N2—N3—Zn1	123.07 (13)	N5—C7—S2	127.03 (16)
C3—N3—Zn1	117.35 (14)	N5—C7—N6	114.16 (19)
C3—N3—N2	119.53 (18)	N6—C7—S2	118.75 (17)
N5—N4—Zn1	120.87 (13)	N6—C8—H8A	109.5
C5—N4—Zn1	117.43 (14)	N6—C8—H8B	109.5
C5—N4—N5	121.53 (18)	N6—C8—H8C	109.5
C7—N5—N4	112.29 (17)	H8A—C8—H8B	109.5
C7—N6—H6	117.1	H8A—C8—H8C	109.5
C7—N6—C8	125.71 (19)	H8B—C8—H8C	109.5
C8—N6—H6	117.1	N7—C9—H9	118.5
C9—N7—Zn1	118.66 (15)	N7—C9—C10	122.9 (2)
C13—N7—Zn1	123.45 (15)	C10—C9—H9	118.5
C13—N7—C9	117.86 (19)	C9—C10—H10	120.8
N1—C1—H1A	109.5	C11—C10—C9	118.4 (2)
N1—C1—H1B	109.5	C11—C10—H10	120.8
N1—C1—H1C	109.5	C10—C11—H11	120.4
H1A—C1—H1B	109.5	C12—C11—C10	119.1 (2)
H1A—C1—H1C	109.5	C12—C11—H11	120.4
H1B—C1—H1C	109.5	C11—C12—H12	120.6
N1—C2—S1	114.89 (16)	C11—C12—C13	118.9 (2)
N2—C2—S1	127.89 (17)	C13—C12—H12	120.6
N2—C2—N1	117.2 (2)	N7—C13—C12	122.8 (2)
N3—C3—C4	124.0 (2)	N7—C13—H13	118.6
N3—C3—C5	114.89 (18)	C12—C13—H13	118.6
			
Zn1—S1—C2—N1	173.89 (15)	N4—N5—C7—N6	178.74 (18)
Zn1—S1—C2—N2	−7.9 (2)	N5—N4—C5—C3	−176.54 (18)
Zn1—S2—C7—N5	17.1 (2)	N5—N4—C5—C6	−0.1 (3)
Zn1—S2—C7—N6	−165.88 (16)	N7—C9—C10—C11	−0.1 (4)
Zn1—N3—C3—C4	177.10 (18)	C1—N1—C2—S1	−179.04 (17)
Zn1—N3—C3—C5	−6.8 (2)	C1—N1—C2—N2	2.6 (3)
Zn1—N4—N5—C7	−15.4 (2)	C2—N2—N3—Zn1	8.8 (2)
Zn1—N4—C5—C3	8.2 (2)	C2—N2—N3—C3	−174.17 (19)
Zn1—N4—C5—C6	−175.37 (16)	C4—C3—C5—N4	175.3 (2)
Zn1—N7—C9—C10	−178.02 (18)	C4—C3—C5—C6	−1.3 (3)
Zn1—N7—C13—C12	178.17 (17)	C5—N4—N5—C7	169.43 (19)
N2—N3—C3—C4	−0.1 (3)	C8—N6—C7—S2	7.9 (3)
N2—N3—C3—C5	175.95 (18)	C8—N6—C7—N5	−174.7 (2)
N3—N2—C2—S1	1.0 (3)	C9—N7—C13—C12	0.2 (3)
N3—N2—C2—N1	179.17 (18)	C9—C10—C11—C12	0.0 (4)
N3—C3—C5—N4	−0.9 (3)	C10—C11—C12—C13	0.2 (3)
N3—C3—C5—C6	−177.50 (19)	C11—C12—C13—N7	−0.4 (3)
N4—N5—C7—S2	−4.1 (3)	C13—N7—C9—C10	0.0 (3)

### Hydrogen-bond geometry (Å, º)

**Table:**

*D*—H···*A*	*D*—H	H···*A*	*D*···*A*	*D*—H···*A*
N1—H1···N5^i^	0.86	2.21	2.988 (3)	150
N6—H6···S1^ii^	0.86	2.65	3.500 (2)	167

Symmetry codes: (i) *x*+1/2, −*y*+3/2, *z*−1/2; (ii) *x*−3/2, −*y*+1/2, *z*−1/2.
