# Supplementary material for: Crystal structure of [butane-2,3-dione bis­(4-methyl­thio­semicarbazonato)-κ4 S,N 1,N 1′,S′](pyridine-κN)zinc(II)
Source: Acta Crystallogr E Crystallogr Commun. 2015 Oct 17;71(Pt 11):1349–51. doi: 10.1107/S2056989015019234 (PMC4645023; doi:10.1107/S2056989015019234)
Supplement: Supplementary file 3 [file e-71-01349-sup3.pdf]

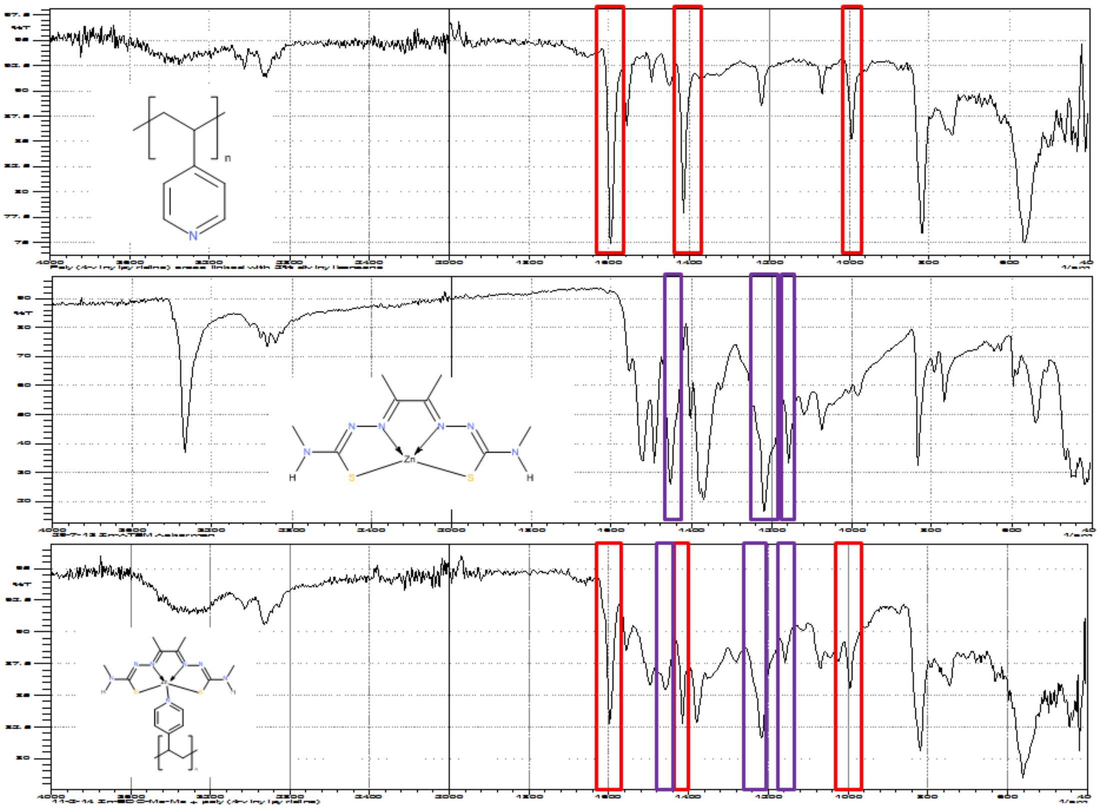

Figure 1. A comparison of the infra-red spectra of poly(4-vinylpyridine), [butane-2,3-dione bis(4-methylthiosemicarbazonato)]zinc(II) and [butane-2,3-dione bis(4-methylthiosemicarbazonato)]zinc(II) on poly(4-vinylpyridine). The red boxes highlight the similar peaks between poly(4-vinylpyridine) and [butane-2,3-dione bis(4-methylthiosemicarbazonato)]zinc(II)on poly(4-vinylpyridine). The purple boxes highlight the similar peaks between [butane-2,3-dione bis(4-methylthiosemicarbazonato)]zinc(II) and [butane-2,3-dione bis(4-methylthiosemicarbazonato)]zinc(II) on poly(4-vinylpyridine).

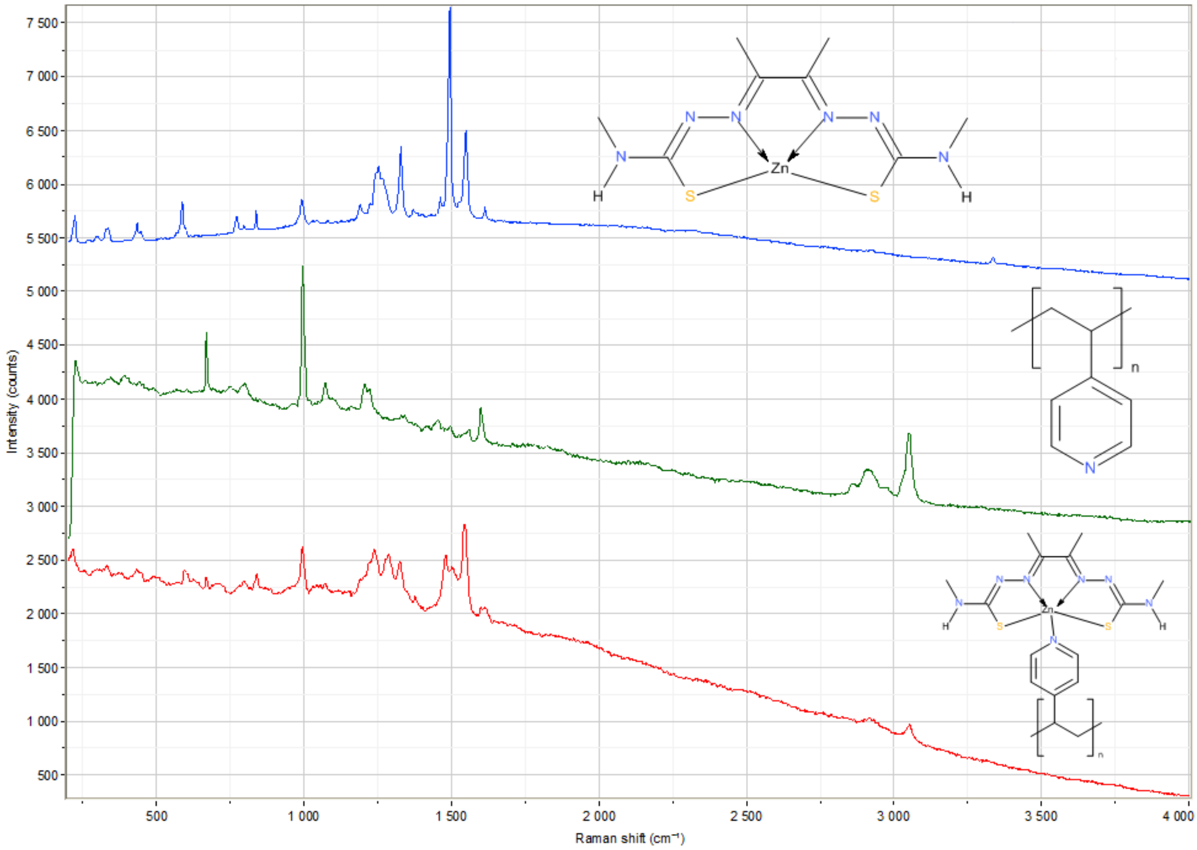

Figure 2. A comparison of the Raman spectra of poly(4-vinylpyridine) (green), and [butane-2,3-dione bis(4-methylthiosemicarbazonato)]zinc(II) (blue) and and [butane-2,3-dione bis(4-methylthiosemicarbazonato)]zinc(II) on poly(4-vinylpyridine) (red).
